# Deductive‐reasoning brain networks: A coordinate‐based meta‐analysis of the neural signatures in deductive reasoning

**DOI:** 10.1002/brb3.1853

**Published:** 2020-09-29

**Authors:** Li Wang, Meng Zhang, Feng Zou, Xin Wu, Yufeng Wang

**Affiliations:** ^1^ Department of Psychology Xinxiang Medical University Henan China; ^2^ Department of Psychiatry Henan Mental Hospital The Second Affiliated Hospital of Xinxiang Medical University Xinxiang China

**Keywords:** activation likelihood estimation, deductive reasoning, meta‐analysis, MRI studies

## Abstract

**Objective:**

Deductive reasoning is a complex and poorly understood concept in the field of psychology. Many cognitive neuroscience studies have been published on deductive reasoning but have yielded inconsistent findings.

**Methods:**

In this study, we analyzed collected data from 38 articles using a recently proposed activation likelihood estimation (ALE) approach and used conjunction analysis to better determine the intersection of the results of meta‐analyses.

**Results:**

First, the left hemispheres in the inferior parietal lobule (Brodmann area 40 [BA40]), middle frontal gyrus (BA6), medial frontal gyrus (BA8), inferior frontal gyrus (BA45/46), caudate, and insula (BA47) were revealed to be significant brain regions via simple‐effect analysis (deductive reasoning versus baseline). Furthermore, IFG, insula, and cingulate (the key neural hubs of the cingulo‐opercular network) were highlighted in overlapped functional connectivity maps.

**Conclusion:**

The findings of the current study are consistent with the view that deductive reasoning requires a succession of stages, which included decoding of linguistic information, conversion and correction of rules, and transformation of inferential results into conclusive outputs, all of which are putatively processed via a distributed network of brain regions encompassing frontal/parietal cortices, as well as the caudate and other subcortical structures, which suggested that in the process of deductive reasoning, the coding and integration of premise information is indispensable, and it is also crucial to the execution and monitoring of the cognitive processing of reasoning.

## Graphical abstract

Deductive reasoning is supported by a distributed network of regions encompassing frontal/parietal cortices and subcortical structures (e.g., caudate). The results of our conjunction analysis highlight the IFG, insula, and cingulate (the key neural hubs of the cingulo‐opercular network) as core locus of deductive reasoning.

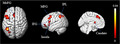

## 1 INTRODUCTION

Deductive reasoning is fundamental to science, human culture, and for deriving solutions to problems in daily life (Fangmeier et al., 2006). The process of deductive reasoning starts with premises and attempts to reach a logically secure conclusion or a series of conclusions from prior beliefs, observations, and/or suppositions that are not explicit in the initial premises. As a higher cognitive activity of human beings, the mental processes underlying reasoning have been the focus of vigorous investigation within psychology and philosophy (Frank et al., 2008; Gordon, 2004).

The advent of neuroimaging techniques has increased the number of studies related to the neural basis of deductive reasoning. Cognitive studies on deductive reasoning have debated whether this process relies primarily on visuospatial mechanisms (Mental Model Theory, MMT) (Johnson‐Laird, 1983) or linguistic models (Mental Logic Theory, MLT) (Rips, 1994), and the experimental results have been inconclusive and inconsistent due to interference from various experimental factors. Some categories of such experimental caveats are as follows:

1. *Mode of the deductive‐reasoning task*. A review by Prado (2011) reported that the engagement of brain regions involved in deductive reasoning depends on the structure of the argument. Relational arguments are associated with activations in bilateral posterior parietal cortex (PPC) and right middle frontal gyrus (MFG), whereas categorical arguments are associated with the left inferior frontal gyrus (IFG) and left basal ganglion (BG). In contrast, propositional arguments are associated with the left PPC, left precentral gyrus (PG), and medial frontal gyrus (MeFG) (Prado et al., 2011).
2. *Complexity of the reasoning task*. Studies have shown that in contrast with simple deductive trials, complex deductive trials result in a pattern of activation across many brain regions, including left dorsolateral prefrontal (Luca et al., 2013), frontopolar, fronto‐medial, left frontal, and parietal cortices (Coetzee & Monti, 2018; Monti et al., 2007).
3. *Stimulus presentation*. Visual experiments have found activity in visual and temporal cortices during the premise processing phase, which suggests that the content for premises elicit visual mental images during this early stage (Fangmeier et al., 2006). However, this pattern of activity is not found in the absence of any correlated visual input; under this circumstance, deductive reasoning activates an occipitoparietal–frontal network, and occipital activation is found in visual association cortex (middle occipital gyrus) but not in primary visual cortex (Fangmeier & Knauff, 2009; Knauff et al., 2002; Markus et al., 2003).
4. *Baseline task*. Knauff et al. (2002) used rest intervals as a baseline for analyzing reasoning of a three‐term series and revealed baseline activation in the left posterior‐temporal cortex. An experiment using an unrelated task (only superficial processing of stimuli) as a baseline task evoked activation of the left IFG, left MFG, temporal gyrus, and cingulate gyrus (Goel et al., 1997, 1998). Moreover, other studies have applied a memory task as a baseline, but the results of these studies have been inconsistent and have been unable to uncover any significant linguistic activations despite these experiments using verbal content (Monti et al., 2007; Monti et al., 2009; Parsons & Osherson, 2001).

A previous qualitative review of the neuroimaging literature on deductive reasoning identified a bilateral but rather left‐centered frontoparietal network for general deductive reasoning across all experiments by multilevel kernel density analysis (MKDA), and subdivided articles into those investigating relational arguments, categorical arguments, and propositional arguments (Prado et al., 2011), which provided a preliminary explanation regarding the differences in results caused by different reasoning arguments. In addition, a recent study incorporated recent research on conditional and syllogistic reasoning based on Prado's research, and further subdivided the article by their structure, content, and requirement for world knowledge, which found a widespread activation network encompassing the frontal, parietal, sublobar, limbic, posterior lobes and exhibit clear distinctions between the task's type and content (Wertheim et al., 2020). However, effective reasoning depends more on the integration of logical relations (i.e., premises) and the application of logical rules in deductive reasoning. Hence, it may be most useful to elucidate brain activation patterns that are independent of the external representational form of deductive reasoning, which would likely delineate the core brain regions underlying the fundamentals of deductive reasoning. In this study, we analyzed collected data using a recently proposed activation likelihood estimation (ALE) approach. The ALE results were assessed against a null distribution of random spatial association between experiments (Eickhoff et al., 2012), and this coordinate‐based meta‐analysis increases the population sample for better generalization by integrating data across several studies. Compared with a previous review (Prado et al., 2011), we innovatively used MACM analyses (meta‐analytic connectivity mapping), which delineates patterns of coactivation across thousands of studies using neuroimaging databases and produces data‐driven functional connectivity maps based on predefined ROIs (Langner et al., 2014). Moreover, MACM allows probing coactivation patterns, that is, task‐based functional connectivity, across a wide range of experimental settings (Bellucci et al., 2018), which may provide a better summary of the deductive reasoning research published over the past few decades from a new perspective.

Despite an increased number of studies on deductive reasoning, results have been inconsistent due to differing experimental conditions across studies. Neuroimaging meta‐analysis combines results of independent experiments to achieve a quantitative summary of the state of research in a specific domain (Turkeltaub et al., 2002). Here, we used ALE meta‐analysis to summarize patterns of activity related to deductive reasoning. Specifically, first, the meta‐analysis results of Prado (2010) have suggested that deductive reasoning does not rely on a unitary brain system but relies on fractionated neural systems located in both cortical (frontal and parietal cortices) and subcortical (BG) structures (Prado et al., 2011). Hence, we collectively analyzed 39 fMRI articles (published over the past 8 years) to further elucidate spatiotemporal brain activation patterns during the processing of deductive reasoning. Second, we used conjunction analysis to better determine the intersection of the results of meta‐analyses, which can further clarify relationships among key brain regions implicated in deductive‐reasoning processing. Third, the interaction between language and thought has become a pivotal phenomenon in the study of human cognition (Frank et al., 2008; Li & Gleitman, 2002), and there has been little agreement regarding this topic. Taken together, this study may further elucidate the relationship between deductive reasoning and language by determining language‐activated brain regions during deductive reasoning, which may serve as indicators of this fundamental cognitive process.

## 2 METHODS

### 2.1 Study selection

**Figure 1:**
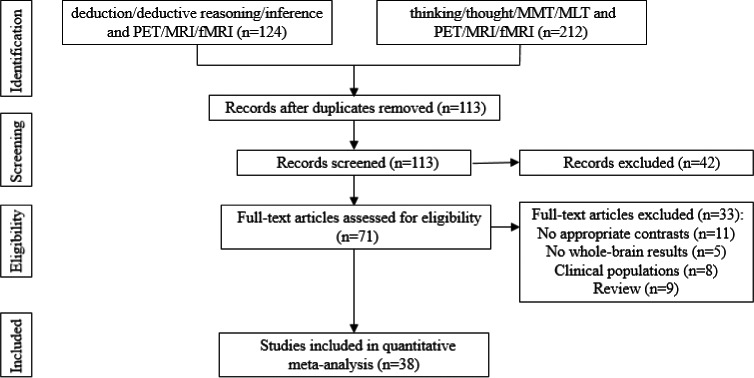
Flowchart of the study selection process for the meta‐analysis

There were two qualitative reviews of the neuroimaging literature on deductive reasoning available prior to our meta‐analysis. One of these reviews (Goel et al., 2007), included studies published through April 2007, while the other review (Prado, 2010) included studies published through September 2010 (on the basis of the previous Goel review). Here, we searched for additional neuroimaging studies of deductive reasoning published from September 2010 to 2019. More specifically, we searched the PubMed, PNAS, SAGE, Oxford Press Wiley, Elsevier Science, and Baidu Scholar databases for studies on several related topics, including the following: reasoning, inference, deduction, deductive reasoning, deductive inference, conditional reasoning, relational reasoning; propositional reasoning, categorical reasoning, thinking, thought, theory of mind, functional magnetic resonance imaging, MRI, fMRI, and PET. Inclusion criteria for the articles were as follows: (a) the paper was written in English; (b) the task in the study involved deductive reasoning; (c) we here used MRI, fMRI, and PET to collect data; (d) all the subjects in the study were healthy without any psychiatric disorders; (e) the coordinates in each of the studies were in the standard Montreal Neurological Institute (MNI) or Talairach space; and (f) all the reported activation coordinates were based on the entire brain. Following these criteria, we ultimately included 38 published, peer‐reviewed fMRI articles on the neural substrates of deductive reasoning in the present meta‐analysis. The specific selection process is shown in Figure 1. From each study, we selected experiments corresponding most closely to a comparison between a reasoning condition and a baseline condition. A summary of the included details of each study in the meta‐analysis is provided in Table 1.

**Table 1:** Studies Included in the meta‐analysis

No.	Studies	Scanning methods	Stimuli modality	*N*	Foci	Conditions
1	Goel et al. (1997)	PET	Visual, linguistic	10	3	Deduction > Baseline
2	Goel et al. (1998)	PET	Visual, linguistic	12	4	Syllogism > Baseline
					5	Spatial relational > Baseline
					5	Syllogism > Spatial relational
3	Osherson et al. (1998)	PET	Visual, linguistic	10	3	Probability > Logic
					8	Logic > Probability
4	Goel et al. (2000)	fMRI	Visual, linguistic	11	13	Syllogism > Baseline
5	Houdé et al. (2000)	PET	Visual, nonlinguistic	8	19	Posttest of conditional reasoning > Pretest
6	Parsons and Osherson (2001)	PET	Visual, linguistic	10	24	Deduction > Probabilistic reasoning
					19	Probabilistic reasoning > Deduction
7	Goel and Dolan (2001)	fMRI	Visual, linguistic	14	19	Relational reasoning > Baseline
					2	Abstract reasoning > Concrete reasoning
					3	Concrete reasoning > abstract reasoning
8	Acuna et al. (2002)	fMRI	Visual, nonlinguistic	15	17	Transitive Inference > Height Comparison Task
9	Knauff et al. (2002)	fMRI	Auditory, linguistic	12	18	Transitive Inference > Baseline
10	Knauff et al. (2003)	fMRI	Auditory, linguistic	12	9	Inference > Baseline
11	Goel and Dolan (2001)	fMRI	Visual, linguistic	16	12	Deductive reasoning > Baseline
12	Goel et al. (2004)	fMRI	Visual, linguistic	14	14	Unfamiliar > Unfamiliar Environment Baseline
					5	Familiar Environment Reasoning > Familiar Environment Baseline
13	Heckers et al. (2004)	fMRI	Visual, nonlinguistic	16	13	Transitive Inference > Baseline
14	Noveck et al. (2004)	fMRI	Visual, linguistic	16	4	MP > Baseline
					6	MT > Baseline
					4	MT > MP
15	Canessa et al. (2005)	fMRI	Visual, linguistic	12	18	Descriptive (DES) conditional reasoning task > Baseline
					23	Social‐exchange (SE) conditional reasoning task > Baseline
					9	Social‐exchange (SE)> Descriptive (DES)
					1	Descriptive (DES)> Social‐exchange (SE)
16	Fangmeier et al. (2006)	fMRI	Visual, nonlinguistic	12	11	Reasoning > Memory
17	Monti et al. (2007) (exp1)	fMRI	Visual, linguistic	10	31	Complex > Simple deductions
18	Monti et al. (2007) (exp2)	fMRI	Visual, linguistic	12	26	Complex > Simple deductions
19	Reverberi et al., 2007	fMRI	Visual, linguistic	14	8	disjunctive > conditional problems integration effect
20	Prado and Noveck (2007)	fMRI	Visual, linguistic	20	10	Complex > Simple conditional reasoning in Verification task
					6	Complex > Simple conditional reasoning in Falsification task
21	Kroger et al. (2008)	fMRI	Visual, linguistic	16	16	logic > math problems
22	Rodriguez‐Moreno et al. (2009)	fMRI	Visual/auditory,linguistic	17	13	Reasoning > Baseline
23	Fangmeier and Knauff (2009)	fMRI	Auditory, nonlinguistic	12	3	Reasoning > Memory
24	Goel et al. (2009)	fMRI	Visual linguistic	17	10	Reasoning > Baseline
25	Monti et al. (2009)	fMRI	Visual linguistic	15	26	Reasoning > Baseline for logic arguments
					43	Reasoning > Baseline for linguistic arguments,
26	Reverberi et al. (2010)	fMRI	Visual linguistic	26	4	Conditional problems > Rest
					9	Syllogistic problems > Rest
					2	Syllogistic problems > Conditional problems
27	Prado et al. (2010)	fMRI	Visual linguistic	15	3	Transitive reasoning task > Rest
					1	Transitive reasoning task > Numerical comparison task
28	Prado and Van Der Henst (2010)	fMRI	Visual linguistic	13	5	integrable arguments > nonintegrable in MT
					7	integrable arguments > nonintegrable in Relational Syllogism
					2	Modus Tollens > Relational Syllogism
					1	Relational Syllogism > Modus Tollens
29	Wendelken and Bunge (2010)	fMRI	Visual, nonlinguistic	16	3	Inference > Baseline
					8	specific relations > general relations
30	Reverberi et al. (2012)	fMRI	Visual linguistic	26	15	Reasoning > Baseline
31	Stollstorff et al. (2012)	fMRI	Visual linguistic	16	2	Incongruent > Congruent in reasoning
					6	Incongruent > Neutral in reasoning
32	Liu et al. (2012)	fMRI	Visual linguistic	14	16	Falsification card > BL
					9	Nonfalsification card > BL
					9	Falsification > Nonfalsification
					13	MT > Nonfalsification
					6	MT > MP
33	Monti et al. (2012)	fMRI	Visual linguistic	21	16	Reasoning > Baseline in linguistic arguments
					10	Reasoning > Baseline in algebraic arguments
					12	algebraic equivalence > linguistic equivalence
34	Prado et al. (2013)	fMRI	Visual linguistic	30	2	verbal task > Rest
					1	spatial task > Rest
35	Cocchi et al. (2014)	fMRI	Visual linguistic	21	18	Rule > null trials
					28	MP > null trials
36	Porcaro et al. (2014)	fMRI	Visual linguistic	13	2	Contradictory > Noncontradictory
37	Alfred et al. (2018)	fMRI	Visual, nonlinguistic	27	4	Reasoning > Baseline
38	Coetzee and Monti (2018)	fMRI	Visual linguistic	20	30	complex > simple reasoning

Note

*N* = number of subjects; Foci = number of coordinates.

### 2.2 Meta‐analysis algorithm

Meta‐analysis was carried out using the revised version (Simon B. Eickhoff et al., 2009) of the ALE approach using Ginger ALE 3.0.2 software (http://brainmap.org/) for coordinate‐based meta‐analysis of neuroimaging results (Laird et al., 2005; Turkeltaub et al., 2002). The key principle behind ALE is to treat the reported foci not as single points, but as centers of three‐dimensional Gaussian probability distributions for capturing the spatial uncertainty associated with each focus (Caspers et al., 2010). The probabilities of all activation foci in a given experiment were combined for each voxel, resulting in a modeled activation map (MA map). Taking the union across these MA maps yields voxel‐wise ALE scores describing the convergence of results at each particular location (Evans et al., 1994). The likelihood of activation for each standard‐space voxel was calculated under a null distribution of spatial independence (Fitzgerald et al., 2010; Sabatinelli et al., 2011). In brief, the ALE algorithm aims at identifying areas showing a convergence of activations across different experiments, and to determine if the clustering is higher than expected under the null distribution of a random spatial association between the results obtained in the experiments.

In the present study, all Talairach coordinates were transformed to MNI space using the icbm2tal transform, which has been shown to provide an improved fit over the mni2tal transform (Brett et al., 2001; Matthew et al., 2002). Based on the MNI stereotactic coordinates reported and transformed by the studies, ALE analysis of single datasets was conducted. ALE maps were created by modeling each focus as a three‐dimensional Gaussian function with a full‐width half‐maximum (FWHM) of 10 mm. Results were thresholded for significance using a cluster‐level family‐wise error (FWE) correction at *p* < .01 with a cluster defining threshold of *p* < .0001 (1,000 permutations, 200 mm^3^ minimum volume) (Eickhoff et al., 2012; Eklund et al., 2016). The results were viewed using MATLAB software (with loaded SPM and DPABI toolboxes) and were overlaid to a standard space using the MNI file.

We defined the brain regions obtained from the meta‐analysis results as our ROIs. To examine the coactivation patterns of these regions commonly recruited by deductive reasoning, we conducted MACM analyses (meta‐analytic connectivity mapping) using the BrainMap Database (http://www.brainmap.org/) (Laird et al. 2009). MACM delineates patterns of coactivation across thousands of studies using neuroimaging databases and produces data‐driven functional connectivity maps based on predefined ROIs (Langner et al., 2014). For our analysis, we constrained our analysis to fMRI and PET experiments from “normal mapping” whole‐brain neuroimaging studies in healthy population, which report activation in standard space. While other studies investigating differences in age, gender, interventions, or clinical populations were excluded. For the IPL, 433 experimental contrasts and 7,041 foci from 5,223 participants were identified; for the MFG, 228 experimental contrasts and 3,372 foci from 3,432 participants; for the MeFG, 422 experimental contrasts and 6,769 foci from 5,140 participants; for the IFG (BA45), 135 experimental contrasts and 1,735 foci from 1,782 participants; for the IFG (BA46), 45 experimental contrasts and 662 foci from 755 participants; for the Caudate, 104 experimental contrasts and 1,897 foci from 1,730 participants; and for the Insula, 116 experimental contrasts and 1,641 foci from 1,708 participants. Importantly, MACM analyses cover experiments in the BrainMap database associated with different types of tasks that involve these activations (Laird et al., 2005). These analyses consisted of the following two steps. First, whole‐brain peak coordinates of all those experiments in the BrainMap database were downloaded if the study reported at least one focus of activation within each ROI. Second, ALE meta‐analyses were conducted over all coordinates of the retrieved experiments to quantify their convergence and coactivation with each ROI. Finally, the ALE maps were family‐wise error (FWE) corrected at a threshold of *p* < .01 at the cluster‐level. The resulting regions were anatomically labeled by reference to probabilistic cytoarchitectonic maps of the human brain using the DPABI toolbox. We reported the peak coordinates of the significant cluster and demonstrated the brain regions nearest the peak coordinates within ± 5 mm (Xin et al., 2015).

## 3 RESULTS

**Figure 2:**
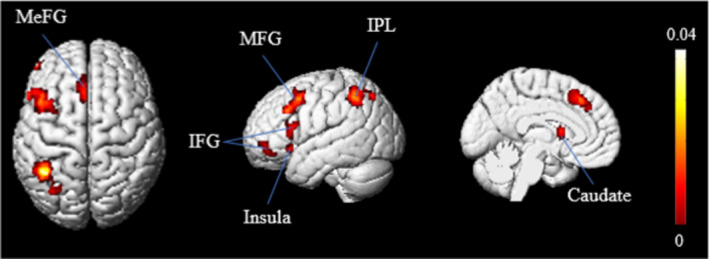
The results of ALE meta‐analysis revealed the key brain regions most consistently activated in neuroimaging studies of deductive reasoning. (IFG, inferior frontal gyrus; IPL, inferior parietal lobule; MeFG, medial frontal gyrus; MFG, middle frontal gyrus)

Selected contrasts from 38 neuroimaging studies of deductive reasoning comparing a reasoning condition and baseline condition yielded a total of 702 foci. Pooling the results of 69 experiments onto a single brain resulted in a diffuse pattern of activation across all lobes, with some clustering evident across left hemispheres in the inferior parietal lobule (IPL, BA 40), middle frontal gyrus (MFG, BA 6), medial frontal gyrus (MeFG, BA 8), inferior frontal gyrus (IFG, BA 45/46), caudate, and insula (BA 47) (Figure 2). Coordinates of the activation maxima of the meta‐analysis on deductive reasoning are provided in Table 2.

**Table 2:** Brain regions that was significantly activated in all deductive‐reasoning studies

Region label	Brodmann area	MNI coordinates		
		*x*	*y*	*z*
L. Inferior Parietal Gyrus	40	−40	−53	49
L. Middle Frontal Gyrus	6	−44	16	44
L. Medial Frontal Gyrus	8	−5	26	45
L. Inferior Frontal Gyrus	45	−53	19	15
L. Inferior Frontal Gyrus	46	−47	47	−8
L. Caudate		−13	6	10
L. Insula	47	−34	21	7

Note

L. left hemisphere.

**Figure 3:**
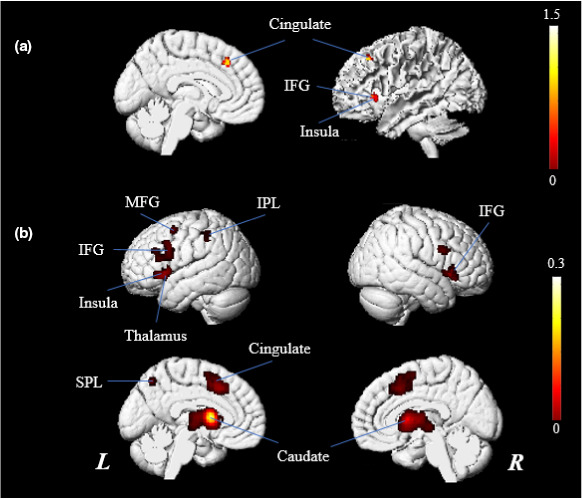
Functional‐connectivity analysis. (a) The results of the analysis for the cortical brain regions of the key brain areas (all brain regions are located in the left hemisphere). (b) The results of the analysis for the caudate (IFG, Inferior Frontal Gyrus; IPL, Inferior Parietal Lobule; L, left hemisphere; MFG, Middle Frontal Gyrus; R, right hemisphere; SPL, Superior Parietal Lobule)

Functional connectivity analysis was conducted to determine the intersection between these key brain regions identified during deductive reasoning. Since the caudate, one of the key brain regions, is a subcortical structure and the other brain regions are cortical structures, two separate conjunction analyses were conducted (one analysis for the cortical brain regions and a separate analysis for the subcortical caudate). The results of the analysis indicated that three common regions existed in the IFG, insula (BA 13), and cingulate gyrus (BA 32) of the left hemispheres among the cortical brain regions. In addition, the bilateral IFG, the bilateral caudate, MFG (BA 6), insula (BA 13), thalamus (BA 32), IPL, superior parietal lobule (SPL), and cingulate gyrus (BA 23/32) of the left hemispheres were revealed as common regions in the conjunction analysis of the caudate (Figure 3). Coordinates of the activation maxima of the results of the conjunction analysis are provided in Table 3.

**Table 3:** Results of the meta‐analysis of functional connectivity

Region label	Brodmann area	MNI coordinates		
		*x*	*y*	*z*
FC analysis for cortical brain regions				
L. Inferior Frontal Gyrus		−36	20	−2
L. Insula	13	−34	20	6
L. Cingulate Gyrus	32	−6	22	40
FC analysis for subcortical caudate				
L. Inferior Frontal Gyrus		−46	14	38
L. Caudate		−14	6	8
L. Middle Frontal Gyrus	6	−32	4	56
L. Insula	13	−32	22	2
L. Thalamus	32	−12	−10	8
L. Inferior Parietal Lobule		−46	−38	44
L. Superior Parietal Lobule		−22	−64	52
L. Cingulate Gyrus		−2	16	44
R. Inferior Frontal Gyrus		34	24	−2
R. Caudate		14	6	6

Note

L. left hemisphere; R. right hemisphere.

## 4 DISCUSSION

Previous studies have claimed that deductive reasoning does not rely on a unitary brain system but relies on a fractionated neural system located in both cortical (frontal and parietal cortices) and subcortical (BG) structures (Prado et al., 2011). As in previous investigations, our ALE meta‐analysis indicated both unified and fractionated neural systems activated in cortical areas, specifically IPL, MFG, MeFG, IFG, and insula, but we also found activity in subcortical (caudate) structures during deductive reasoning. On the whole, all the brain regions that were significantly activated were located in the left hemisphere, which is consistent with findings from previous studies on the neural basis of deductive reasoning. Monti et al. (2007) revealed a content‐independent network that was responsible for carrying out deductive processes and included the left hemisphere (frontal and parietal cortices). Moreover, experiments with patients suffering from brain lesions in the prefrontal lobe (Goel et al. 2007), temporal cortex (Langdon & Warrington, 2000; Read, 1981), or throughout widespread regions across the entire hemisphere (Golding, 1981) have supported a left hemisphere dominance for deductive reasoning. One possible explanation for this spatial localization is that when confronted with the premises of a deductive argument, the left hemisphere might recognize logical structures and generate a hypothesis regarding a collective conclusion (Goel, 2007). It is not surprising to link mental model theory, having a visuospatial nature, with right hemisphere activation and mental logic theory—which have a propositional nature—with left hemisphere activation (Heit, 2015). Nevertheless, some researchers (e.g., Knauff et al., 2002) have suggested that left hemisphere activation may be consistent with mental model theory, because comprehension of arguments will recruit linguistic areas of the brain (Monti et al., 2007, 2009). However, intrahemispheric differences are apparent, and we will discuss relevant regions in the following sections:

For cortical (IPL, IFG and insula) structures, some studies have identified that the IPL is involved in spatial realignment, which governs shifts of spatial attention and target detection (Chapman et al., 2010; Shulman et al., 2010). Likewise, a large body of evidence coming from neuroimaging studies has implicated the activation of the left IFG in syntactic processing at the word or sentence level (Friederici & Kotz, 2003; Grodzinsky & Santi, 2008), which appears to support a syntactic‐ or rule‐based view of deductive reasoning (Goel. 2007). More specific, the left IFG (BA 45) is activated during both the encoding and the integration of propositional premises, and the BA46 of the frontal cortex is involved in the control and allocation of attention (Bishop, 2009; Luks et al., 2002), Others have also found that BA46 (Dorsolateral prefrontal cortex) may represent the site of meta‐cognition (Nelson, 1990; Tzu‐Ching et al., 2014), and is associated with rule‐guided operations in the early stage of conditional‐proposition testing (Jimei et al., 2012). Additionally, it is noteworthy that both the IPL and IFG are canonical mirror neuron brain regions in humans (Buccino et al., 2004; Chong et al., 2008; Iacoboni et al., 1999; Kilner et al., 2009), which play a crucial role in associative learning (Heyes, 2010; Ray & Heyes, 2011). Associative learning is often considered as a stable adaptation for tracking relationships between events (Heyes, 2012), and the mirror neuron system plays an important role in the representation and storage of simple and complex relationships (Cook, 2012; Ferrari & Fogassi, 2005; Keysers et al., 2003). Here, the IPL and IFG may be closely related to the activation of logically related experiences, which may indicate that human deductive reasoning is at least partially derived from experience/schema/mental models and that both of these two brain regions might mediate conclusions or activities in the context of processing premises. In other ways, the IPL and IFG—as key components of the mirror neuron system—may help us understand people's actions, words, and intentions, and it's even an important brain region for human empathy (Baird et al., 2011; Hojat & Cohen, 2012), which seem to a certain extent that the human reasoning system is an evolutionary system that helps individuals to prove and justify on the basis of understanding external information during evolution or social activities. Moreover, The activation of the insula seems to confirm the participation of higher‐order cognitive control and attention processing during this process (Luca et al., 2013).

Furthermore, the MFG and MeFG have been connected to working memory in previous studies (Kirschen et al., 2005; Koechlin et al.; Owen, 2010; Ranganath et al., 2003; Ricciardi et al., 2006). An early response in the left MFG potentially reflects semantic comprehension processing (Porcaro et al., 2013). In this context, the MFG and MeFG may be particularly sensitive to the difficulty of deductive reasoning.

For subcortical (caudate) structures, previous imaging studies of reasoning have reported that activation of the caudate is involved when multiple rules need to be deduced and integrate (Christoff & Prabhakaran, 2001; Fangmeier et al., 2006; Prabhakaran et al., 2001). Furthermore, caudate activation has been linked to planning and executing demands of reasoning in both reasoning and working memory systems (Melrose et al., 2007). Moreover, in the present study, we found that the frontal lobe (IFG and MFG), parietal lobe (SPL and IPL) cingulate gyrus, insula, caudate and thalamus were all significant regions as indicated by conjunction analysis of the caudate. About frontal lobe, IFG and MFG are mainly related to the representation and integration of premise information and working memory. Similar to the IPL, left SPL occurs across a variety of tasks requiring manipulation and rearrangement of information in spatial working memory (Koenigs et al., 2009) and allocation of spatial attention (Dehaene et al., 2003). In regard to the cingulate gyrus, several recent neuroimaging findings suggest that it is associate with general executive, and it also plays a central role in a wide spectrum of highly integrated tasks such as visuospatial imagery and episodic memory retrieval (Cavanna & Trimble, 2006; Sommer et al., 2010). More significantly, the dorsal anterior cingulate and insular cortices represent key neural hubs of the cingulo‐opercular network, which is implicated in maintaining and implementing task set (Dosenbach et al., 2006, 2007). Additionally, Many previous studies have indicated that the thalamus is related to various higher brain functions such as memory and learning (Karussis et al., 2000; Nagaratnam et al., 1999; Radanovic & Scaff, 2003), and may be related to the regulation and/or facilitation of ongoing cortical processing of memory and language (Baker et al., 1997; Ojemann et al., 2015; Elizabeth et al., 1996; Warburton et al., 1996). As an important nucleus within the basal ganglia, the caudate nucleus has a strong connection with the dorsolateral prefrontal lobe, and its interaction with the prefrontal lobe in deductive reasoning task may be related to the facilitation of inferences and application of rules.

The results of our functional conjunction analysis among the four cortical brain regions indicated that three common regions existed in the IFG, insula (BA 13), and cingulate gyrus (BA 32) of the left hemispheres. According to the brain maps, the activation position of the inferior frontal gyrus is adjacent to the triangle (BA45) (close to insula), and inferior frontal gyrus pars triangularis is a part of the frontoparietal operculum, which covers the upper surface of the insula. Reverberi et al. (2010) demonstrated that the left IFG (BA 45) is activated during both the encoding and the integration of propositional premises, and this region also has been suggested to be involved in the extraction and representation of the superficial structure of a problem (Reverberi et al., 2007, 2010). About insula, recent evidence from network analysis suggests a critical role for the insula in high‐level cognitive control and attentional processes, and more researches unveiled that the insula is unique in that it is situated at the interface of the cognitive, homeostatic, and affective systems of the human brain, providing a link between stimulus‐driven processing and brain regions involved in monitoring (Luca et al., 2013; Menon & Uddin, 2010). In addition, the neuroimaging results converge with the general executive, learning, and conflict resolution processes in anterior cingulate cortices (BA 32) (Botvinick et al., 1999, 2004). Beyond that, the dorsal anterior cingulate and insular cortices represent key neural hubs of the cingulo‐opercular network, which is implicated in maintaining and implementing task set (Dosenbach et al., 2006, 2007). Compare to previous findings, our present meta‐analysis revealed that the IFG, insula, and cingulate may represent potential core site to collaborate in deductive reasoning. However, several studies have shown that the left IFG (BA44), which largely overlaps with the traditional Broca's area, is a key area in representing the formal structure of a logical problem during deductive reasoning (Reverberi et al., 2012; Reverberi et al., 2009). Here, our IFG cluster was positioned on is adjacent to the triangle (BA45) and closer to insula rather than BA 44 corresponding to Broca's Area. Besides, as mentioned above, the functions of the insula and cingulate are related to high‐level cognitive control, execution, and monitoring, which suggested that in the process of deductive reasoning, the coding and integration of premise information is indispensable, and it is also crucial to the execution and monitoring of the cognitive processing of reasoning.

## 5 CONCLUSION

Overall, our results suggest that deductive reasoning is supported by a distributed network of regions encompassing frontal/parietal cortices and subcortical structures (e.g., caudate), and the results of our conjunction analysis highlights the IFG, insula, and cingulate (the key neural hubs of the cingulo‐opercular network) as core locus of deductive reasoning. These results conflict with the supposition that logic is subserved by a set of language‐independent regions within frontopolar (BA10) and fronto‐medial (BA8) cortices related to the putative core of deductive reasoning (Monti et al., 2007, 2009). Moreover, a recent study revealed that the transient inhibition of Broca's area disrupted linguistic processing without affecting thought processing (Coetzee & Monti, 2018). The discrepancies between these results support the view that deductive reasoning requires a succession of stages that progressively transform premises into a conclusion or a series of conclusions; these stages include the decoding of the initial linguistic information, the conversion and correction of rules, and the transformation of inferential results into conclusive outputs (Coetzee & Monti, 2018; Monti et al., 2009). However, our study did not have valid classification criteria to sufficiently separate these different stages of deductive reasoning. Hence, future research can use high‐time resolution EEG and NMR techniques to synchronously record the brain signals of participants or inhibit the activity of certain brain regions to further elucidate the activation characteristics of brain regions at different stages of deductive reasoning and the precise relationship between deductive reasoning and linguistic representations. Besides, researchers need to increase the diversity of reasoning materials. in addition to abstract materials, we should also increase concrete materials that exist in real life to help us better understand reasoning.

## CONFLICT OF INTEREST

The authors declared that they had no conflicts of interest with respect to their authorship or the publication of this article.

## AUTHOR CONTRIBUTIONS

L. Wang and M. Zhang developed the study concept. Study Selection and Meta‐analysis algorithm were performed by all authors. L. Wang and M. Zhang drafted and revised the manuscript. All authors approved the final version of the manuscript for submission.

### Peer Review

The peer review history for this article is available at https://publons.com/publon/10.1002/brb3.1853

## Data Availability

The data that support the findings of this study are available from the corresponding author upon reasonable request.
